# The North-Western Italian experience with anti IL-5 therapy amd comparison with regulatory trials

**DOI:** 10.1186/s40413-018-0210-7

**Published:** 2018-12-06

**Authors:** Diego Bagnasco, Manlio Milanese, Giovanni Rolla, Carlo Lombardi, Caterina Bucca, Enrico Heffler, Giorgio Walter Canonica, Giovanni Passalacqua

**Affiliations:** 1 Allergy and Respiratory Diseases, IRCCS Policlinico San Martino, University of Genoa, Genoa, Italy; 2 Division of Pneumology, S.Corona Hospital, Pietra Ligure, Italy; 3 Allergy and Immunology, AO Mauriziano Hospital, University of Turin, Turin, Italy; 4 Departmental Unit of Allergology & Respiratory Diseases, Fondazione Poliambulanza, Brescia, Italy; 5 Azienda Ospedale-Università Città della Salute e della Scienza, S.C. Pneumologia, Turin, Italy; 6 Personalized Medicine Clinic Asthma and Allergy, Humanitas Clinical and Research Center, Department of Biomedical Sciences, Humanitas University, Rozzano, Milan, Italy

**Keywords:** Severe asthma, Uncontrolled asthma, Eosinophilic asthma, Mepolizumab, IL-5, Real-life

## Abstract

**Background:**

The severe forms of asthma represent a major burden, because of severity of symptoms, costs and impact on everyday life. Recently, Mepolizumab (MEP) was approved and marketed for the treatment of hypereosinophilic severe asthma. This anti-IL-5 monoclonal antibody reduced exacerbation rates and oral corticosteroid (OCS) use in well selected patients. The aim of this study was to evaluate the characteristics of patients receiving MEP in a real-life setting. Thus, we describe a retrospective analysis of patients treated with MEP in six centres in North Western Italy, including those who participated in the main regulatory trials.

**Methods:**

The baseline data, before prescription, from six North Western Italy severe asthma clinics, between June 1st 2017 and December 31st 2017, were evaluated. The collected real-life data were then compared with those of SIRUS, MENSA, DREAM and MUSCA trials.

**Results:**

Sixty-five patients were included (45% female; mean age 56 years; age range 19–84). Main observed differences with regulatory trials could be observed in eosinophils blood count at baseline, where the mean of our real-life patients (653 cells/μL) was overall higher than the one of all trials (240 cells/μL, 296 cells/μL, 253 cells/μL; *p* <  0.0001). The incidence of polyposis was also significantly higher in our sample (72% vs. 24%, 49%, 10%, 19%; p <  0.0001). The daily average dose of OCS was lower in our real-life patients (9 mg), if compared with SIRIUS (13.7 mg), MENSA (13.2) and MUSCA (13), and similar to the data published in DREAM (10.8).

**Conclusions:**

The comparison of real-life patients' characteristics with regulatory trials, displayed several apparent discrepancies. The demographic and clinical aspects were similar in all groups, whereas other features (eosinophil count, pulmonary function FEV1%) differed. These data, for the first time, could represent a basis for a more accurate prescription of the drug.

## Background

Asthma is a common chronic respiratory disease, which prevalence ranges between 1 and 18%, with slight variations among Countries, and according to selection criteria and methodology [1]. The severe form affects about 5–10% of asthmatic patients. According to guidelines of the American Thoracic Society and European Respiratory Society (ATS/ERS) [2], a “severe” patient needs a maximal therapy (step 4–5 GINA) [3] with the chronic use of systemic steroids, usually oral corticosteroids (OCS). To provide a different therapeutic approach to the more severe forms of asthma, biological treatments targeting the known pathogenic mechanisms have been hypothesized and realized. The first commercialized biological treatment was Omalizumab (an anti-IgE antibody) [4]. Recently, an IL-5 antagonist was commercialized, mepolizumab (MEP), directed to circulating IL-5. Its effectiveness in hypereosinophilic asthma is now well ascertained [5]. In clinical trials MEP displayed a favourable efficacy in reducing exacerbations, OCS intake, and in enhancing the quality of life (QoL). It appeared that a careful selection of patients is needed to achieve the best results. In fact, in the earliest trial MEP was given to a non-selected population of patients with moderate persistent asthma and variable levels of eosinophils. No significant clinical result emerged for asthma control and specific bronchial hyperresponsiveness [6, 7]. Subsequently, other big trials including patients with at least 150–300 eosinophils/mmc) were performed, with favourable results [8–11]. Following the commercial release of MEP, we attempted to strictly evaluate the characteristics of the treated patients in a real-life setting, as compared to regulatory trials. In this retrospective we provide a description of the characteristics of patients treated with MEP, in six centres in the North-Western Italy, including those who participated in the main clinical trials.

## Methods

This is a retrospective analysis of patients from six severe asthma clinics of North West Italy (IRCCS Policlinico San Martino Genoa, Santa Corona Hospital Pietra Ligure, AO Mauriziano, AOU Città della Salute e della Scienza Turin, Humanitas University Milan, and Fondazione Poliambulanza Brescia), which were the first clinics to have the product available. All patients meet the ATS/ERS criteria for uncontrolled severe asthma [2] and the conditions to be prescribed with MEP according to the Italian Drug Agency. A documented blood eosinophilia of at least 300 cells/mmc in the previous year, a current count of at least 150 cells/mmc and at least 2 cycles of OCS during the previous year, or continuous OCS therapy [12] were required. All patients started MEP treatment between June 1st, 2017 and December 31st, 2017, at the dose of 100 mg subcutaneously every month. The data of the considered patients were matched with those of SIRIUS, MENSA, DREAM and MUSCA [8–11] studies.

All procedures described herein were performed as per standard of care. The study was observational, with approved and commercialized drugs. No approval from the Ethics Committees was required according to the Italian laws. The privacy rules were applied and all patients provided an informed consent to be treated.

### Statistical analysis

All patients receiving MEP in the aforementioned period were included in the analysis. Arithmetic mean and standard deviation were used for the descriptive statistics. To compare data of our patients with those of the four mentioned clinical randomised, double-blind, placebo-controlled studies [8–11], a weighted average was used due to the difference in sample sizes. To compare our data with those of the main clinical trials with MEP we used a *t-test one sample* and a *Z-test one proportion* when indicated. A *p* value of ≤0.05 was considered to be significant.

## Results

### General description

Starting from June 1st, 2017 to December 31st, 2017, 65 severe hypereosinophilic asthmatic patients were treated. All patients were classified as step 4/5 GINA [3], remaining uncontrolled despite the maximal therapy [2]. The mean age was 56 ± 11.5 (range 19–84) (Table 1). Seven patients (11%) had their asthma symptoms before adolescence. Mean age of onset of asthma was 38 ± 16.5 (range 2–68), with a mean duration of the disease, before MEP treatment of 18.2 years (±14.4). Females accounted for 45% of the whole population. 17/65 patients (26%) were former or current smokers.

**Table 1:** The current observed population receiving MEP

		SD or percentage
Number of patients	65	–
Female/Male	29/36	45% female
Age range	19–84	–
Age mean	56	±11.5
Current smoker	17	26%
Duration of asthma (years)	18.2	± 14.4
Historical eosinophilic count	1046	± 885
Blood eosinophil count at baseline	653	±381
Mean exacerbations previous12 months	3.0	±1.8
Hospitalized patients previous 12 months	20	31%
Mean OCS dose at baseline (mg)	9.2	±9.2
Mean FEV_1_ at baseline (%)	73	±18.1
ACT	16.6	±4.7
Concomitant nasal polyposis	47	72%

The control of the disease was evaluated with validated asthma control test (ACT) at baseline with a mean value of 16.6 ± 4.7 (range 6–25). Also lung function test prior to MEP evidenced a mean forced expiratory volume at the first second (FEV_1_) of 73 ± 18%. Fifty-three out of 65 patients (82.5%) were receiving daily OCS therapy at the time of inclusion, with a mean dose of 9.2 mg (±9.2) of prednisolone (different molecules were converted in prednisolone equivalent). The mean eosinophils level at baseline, before treatment, was 653 cells/mmc (range 150–1987), with a mean historical value in the previous year of 1046 cells/mmc (range 320–6000). Nasal polyps were documented (by fiberoptic rhinoscopy and/or computerized tomography) in 47 (72%) subjects. 7/65 (11%) patients come from the previous regulatory trials conducted at our centres.

### Comparison with the clinical trials (Table 2)

The mean age in our real-life sample was 56 years, with a statistically significant difference compared to MUSCA (51 years; *p* <  0.0005), DREAM (46 years; *p* <  0.0001), MENSA and SIRIUS (50; p <  0.0001). Concerning gender, MEP was given to 29/56 women (45%) in the real-life sample. A statistically significant difference towards the four examined trials was seen with the DREAM study (387/621 *p* = 0.0033) and the MUSCA study (325/551, 58% *p* = 0.04). Concerning smoking habit, in our sample 17/65 (26%) were current or former smokers at baseline. If in DREAM, MENSA and MUSCA study the percentage of smoking people was similar to which observed in our sample, the same cannot be said about the SIRIUS study, where the percentage of smokers was higher (39%; *p* = 0.0459).

**Table 2:** Comparison among groups. Real-life population and regulatory studies. The significant difference for the available parameters are indicated in boldface

		CURRENT POPULATION	SIRIUS (11)	MENSA(10)	DREAM (8)	MUSCA (9)
Age	Mean ± SD (range)	56 ± 11.5 (19–84)	50 (*n.p.*) (16–74)	50 (*n.p.*) (12–82)	46 ± 11.2 (*n.p.*)	51 ± 13.4 (*n.p.*)
	*P*-value	–	**< 0.0001**	**< 0.0001**	**< 0.0001**	**< 0.0001**
Female %	*N* (%)	29 (45)	27 (55)	329 (57)	392 (63)	325 (58)
	*P*-value	–	n.s	n.s	**0.003**	**0.039**
Duration of asthma (years)	Mean ± SD	18.2 ± 14.4	18.27 ± 13.1	19.9 ± 13.8	19.1 ± 45.8	19.5 ± 14.8
	*P*-value	–	n.s.	n.s	n.s	n.s
Smoker	*N* (%)	17 (26)	53 (39)	159 (28)	133 (22)	147 (26)
	*P*-value	–	**0.04**	n.s.	n.s	n.s
FEV_1_%	Mean ± SD	73 ± 18.1	58.7 ± 17.7	61 ± 17.9	60 ± 16	55 ± 14.5
	*P*-value	–	**< 0.0001**	**< 0.0001**	**< 0.0001**	**< 0.0001**
Eosinophils baseline (cells/μl)^a^	Mean ± SD *N* (%)	653 ± 381 ≥150 = 64 (98) ≥300 = 9 (91)	240 ± 1126 (*n.p.*) (n.p.)	296 ± 992 (*n.p.*) (n.p.)	253 ± 1022 (*n.p.*) n.p.)	325 (*n.p.*) ≥150 = 474 (86)^b^ ≥300 = 351 (64)^b^
	*P*-value	–	**< 0.0001**	**< 0.0001**	**< 0.0001**	**0.0043** ^b^ **< 0.0001** ^b^
Exacerbations/ 12 months	Mean ± SD	3 (1.8)	3.1 (3.1)	3.6 (2.6)	3.58 (3.03)	2.8 (1.7)
	*P*-value	–	n.s.	**0.0047**	**0.0087**	n.s.
ER access/ 12 months	*N* (%)	20 (31)	23 (17)	109 (19)	150 (24)	179 (32)
	*P*-value	–	**0.005**	n.s	n.s	n.s
OCS dose at baseline	Median (range) Mean (SD)	9 (0–50) 9.2 ± 9.2	13.7 (5–35) (*n.p.*)	13.2 (1–80) (*n.p.*)	10.8 (8–20) (*n.p.*)	(*n.p.*) 13 (±10.9)
	*P*-value	–	**0.0002**	**0.0007**	n.s.	**0.0014**
Nasal polyposis	*N* (%)	47 (72)	33 (24)	281 (49)	62 (10)	105 (19)
	*P*-value	–	**< 0.0001**	**< 0.0001**	**< 0.0001**	**< 0.0001**

^a^Geometric mean, *n.p* not provided, ^b^comparison in % of patients, *n.s* not significant

The duration of asthma was also considered, calculated as the difference between the date of first MEP administration and the reported first occurrence of asthma symptoms. The range spans widely from 2 to 68 years, but only 7 patients complained of symptoms before the age of 20 years. The average age of onset of asthma was 38 years, with a mean duration of 18.2 (±14.2) years. In this context our data parallel those collected in the main clinical trials. Pulmonary function was evaluated according to Global Lung Function Initiative (GLI) predictions [13], and the mean percentage of FEV_1_ was 73 ± 18.1%. Also in this case, the observed values were different from those of clinical trials, where the weighted averages were 58.7 (±17.7) in SIRIUS, 61 (±17.9) in MENSA, 60 (±16.0) in DREAM and 55 (±14.5) in MUSCA, all value with a statistically difference <  0.0001. The range of eosinophils, in the previous and current years, was broadly distributed with a mean value of 1046 (range 320–6000) cells/μl and 653 (range 150–1987) cells/μl, respectively. Matching these results with those of previous clinical trials, a mean value of 653 (±381) cells/μl was recorded in our cohort, whereas in the four randomized trials the concentration of eosinophils at baseline (SIRIUS 240 cells/μl, MENSA 296 cells/μl, DREAM 253 cells/μl and 325 for MUSCA) was lower, all with a statistically significant difference (*p* <  0.0001). Comparing our data with that of the MUSCA study, where eosinophils were categorized in two ranges, we observed a statistically significant difference in both groups (98% vs 86%, *p* = 0.0043 in ≥150 cells/μL; 91% vs. 64%, *p* <  0.0001 in ≥300 cells/μL). The mean exacerbation rate of our population was 3.0 (±1.8), not different from of SIRIUS (±3.1; *p* = 0.5644), MUSCA (±1.7; *p* = 0.4561), and lower than in MENSA (3.6 ± 2.6; *p* = 0.0047) and DREAM (3.58 ± 3.03; *p* = 0.0087). The percentage of subjects hospitalized or requiring emergency room visit, was higher in our sample (31%) as compared to SIRIUS (17%; *p* = 0.005) and MENSA (19%; *p* = 0.024), similar in MUSCA (32%; *p* = 0.94) and lower in the DREAM trial (24%; *p* = 0.26).

The use of OCS is a main determinant in severe asthma. Among our real-life patients, 53 had a daily prescription of OCS (5–50 mg of prednisone or equivalent, mean value 9.2 mg). Only in the DREAM study the daily dose was similar to our patients, (10.76 mg; *p* = 0.1715), in SIRIUS (13.7 mg; *p* = 0.0002), MENSA (13.2 mg; *p* = 0.0007) and MUSCA (13 mg; *p* = 0.0014) trials. The daily dose was on average higher than in our sample. Finally, ACT was somewhere used to evaluate the QoL, but this aspect was difficult to compare, since different instruments were used in the different studies. When the presence of nasal polyps was assessed by endoscopy [14], in the real-life population the percentage of patients with nasal polyps was surprisingly highest than in the reference trials: 72% in real life, 49% in MENSA (*p* = 0.0003), 19% in MUSCA (*p* <  0.0001) and 10% in DREAM (*p* <  0.0001). No data were available from the SIRIUS study. This may be explained by the strict inclusion criteria of the regulatory trials that were not applied to our real-life patients. Also, it has to be considered that the association of polyposis, asthma and hypereosinophia is quite frequent, and usually underdiagnosed [15].

## Discussion

Our clinics began to use MEP in the real-life setting, since the drug was approved and released by the local regulatory agency. Thus, we attempted to evaluate the characteristics of eligible patients, and to compare the data to those obtained from the major regulatory-oriented trials. The data herein collected in real-life setting confirm that eosinophilic asthma is predominantly a disease of adulthood (late onset asthma) [16], as compared with the early onset form that is most frequently characterized by an allergic-mediated pattern. At variance with omalizumab, where FEV_1_ below 80% of predicted was mandatory to start the add-on treatment in severe uncontrolled asthma [17], no limitation in lung function was recommended to prescribe MEP [18].

With the commercialization of MEP severe uncontrolled hypereosinophilic asthma certainly achieved more expanded therapeutic improvement. The clinical data about efficacy (reduction of exacerbations, decrease in OCS use, improved QoL) together with the safety profile are promising. It is true that, in comparison with the regulatory trials, in real life, the high level of blood eosinophils count is essential to decide for the IL-5 antagonism strategy. The possibility of decreasing the OCS burden would be regarded as a relevant socio-economical advantage [19].

Nevertheless, this observational trial evidenced some discrepancies between real life patients and clinical trials. The age at first administration and gender are probably not relevant, whereas the presence of eosinophils blood count at baseline or the presence of nasal polyposis are of importance. Also, it seems from the real-life observation that a more precise judgement would require at least 1 year of treatment.

One of the main endpoints of all biological drugs remains, in severe asthma, the reduction of the use of OCS or inhaled corticosteroids [19] and in this context, the use of MEP produced a significant reduction in OCS usage. Although randomized controlled trials (RTCs) play a pivotal role in the experimental development plan of a drug [20–23], and its subsequent clinical use, often the characteristics of the patients enrolled in the registration clinical trials do not reflect what happens in real-life.

## Conclusions

We report herein the characteristics of a real-life patient population compared to the “formal” trials, showing some apparent discrepancies among the populations. In general, the demographic and clinical aspects were similar among the patient groups, but the groups themselves displayed some differences that could be taken into account for a more refined definition of the appropriate prescription.

## Abbreviations

ACT: Asthma Control Test
FEV1: Forced expiratory volume at the first second
MEP: Mepolizumab
OCS: Oral Corticosteroids
QoL: Quality of life
